# Outcome of 449 adult patients with rhabdomyosarcoma: an observational ambispective nationwide study

**DOI:** 10.1002/cam4.1374

**Published:** 2018-06-28

**Authors:** Emmanuelle Bompas, Loïc Campion, Antoine Italiano, Axel Le Cesne, Christine Chevreau, Nicolas Isambert, Maud Toulmonde, Olivier Mir, Isabelle Ray‐Coquard, Sophie Piperno‐Neumann, Esma Saada‐Bouzid, Maria Rios, Jean‐Emmanuel Kurtz, Corinne Delcambre, Pascale Dubray‐Longeras, Florence Duffaud, Marie Karanian, François Le Loarer, Patrick Soulié, Nicolas Penel, Jean‐Yves Blay

**Affiliations:** ^1^ Institut de cancérologie de l'ouest – René Gauducheau Saint Herblain France; ^2^ Institut Bergonié Bordeaux France; ^3^ Institut Gustave Roussy Villejuif France; ^4^ Institut Universitaire du Cancer Toulouse– Oncopole Toulouse France; ^5^ Centre Georges‐François Leclerc Dijon France; ^6^ Centre Léon Bérard Lyon France; ^7^ Institut Curie Paris France; ^8^ Centre Antoine Lacassagne Nice France; ^9^ Institut de Cancérologie de Lorraine Vandoeuvre‐les‐Nancy France; ^10^ Hôpitaux universitaires de Strasbourg Strasbourg France; ^11^ Centre François Baclesse Caen France; ^12^ Centre Jean Perrin Clermont‐Ferrand France; ^13^ CHU de Marseille Marseille France; ^14^ Institut de cancérologie de l'ouest – Paul Papin Angers France; ^15^ Centre Oscar Lambret Lille France

**Keywords:** Adult cancer, pediatric, rhabdomyosarcoma

## Abstract

Five‐year overall survival (OS) of localized RMS exceeds 70% in children (<18) but is very poor in adult patients. We analyzed the outcome and prognostic factors (PF) of a national series of adult patients with RMS in a large study. The study population consisted of two different cohorts: a retrospective cohort (157 adult patients treated in 13 reference centers between 05/1981 and 02/2010) and the prospective cohort (292 patients with RMS diagnosed and treated between 01/2010 and 12/2014 in France) included in the NetSarc database. A descriptive analysis of patients’ characteristics and prognostic factors was conducted on both series which were compared. In the retrospective series, histological subtypes were embryonal (E‐RMS) for 21% of patients, alveolar (A‐RMS) for 35% of patients, and “adult‐type” P‐RMS (pleomorphic, spindle cell RMS, not otherwise specified) (P) for 44% patients. This distribution significantly differed in the prospective cohort: A‐RMS: 18%; E‐RMS: 17%; and P‐RMS 65%. With a median follow‐up of 8.5 years, 5‐year OS for localized RMS and advanced RMS (with nodes and/or metastases) was 43% and 5%, respectively, (*P* < 0.0001), and median OS was 51, 33, and 16 months for E‐RMS, A‐RMS, and P‐RMS, respectively, in the retrospective cohort. The median OS was less than 40 months for the prospective nationwide cohort for the entire population. In a multivariate analysis of the retrospective study, independent prognostic factors for OS were A‐RMS, R0 resection, and adjuvant radiotherapy (RT). For localized RMS, age and use of pediatric chemotherapy (CT) regimen are independent prognostic factors. Adult patients with RMS have a poorer overall survival than pediatric patients, and survival varies considerably across histological subtypes.

## Introduction

Rhabdomyosarcoma (RMS) accounts for <3% of adult soft tissue sarcoma but is the most frequent soft tissue sarcoma histological subtype before age 10 and the 4th most prevalent cancer during childhood 1, 2, 3, 4. Five‐year overall survival (OS) of children has dramatically improved in the last 30 years based on the results of successive studies of large multinational collaborative trials dedicated to children. Currently, the 5‐year OS exceeds 70% for nonmetastatic RMS 5, 6, 7, 8, 9, 10. In contrast, the outcome of adult patients remains poor. Furthermore, given the rarity of adult RMS, limited information is available in the literature, and optimal management and identification of prognostic factors (PF) have not been established in large cohorts, in particular in nationwide studies 11, 12, 13, 14.

The primary aim of the present retrospective study was to describe the treatment, outcome, and prognostic factors for adult patients with RMS. We analyzed data from a retrospective database of patients treated in reference centers as well as a more recent prospective nationwide database.

## Patient and Methods

### Retrospective study

The retrospective study was conducted using the “Conticabase” (see http://www.conticabase.org), which was described elsewhere (https://www.ncbi.nlm.nih.gov/pubmed/27207359). The data were obtained from 13 reference centers that managed incident cases of adult RMS between May 1981 and February 2010. A total of 157 adult patients (>18 years) with RMS were included. Histological diagnosis was confirmed with a central review by expert pathologists of the French Sarcoma Group (FSG). According to WHO classification, histological subtypes included embryonal RMS (E‐RMS), alveolar RMS (A‐RMS), or “adult‐type” RMS (including pleomorphic, adult spindle cell RMS, and not otherwise specified RMS: P‐RMS). Histological grade was established using the FNCLCC grading.

### Prospective study

The data collection process in the prospective study is described elsewhere (see Netsarc.org; https://www.ncbi.nlm.nih.gov/pubmed/26974708). This prospective cohort included 292 adult patients with RMS (>18 years) treated in France in reference centers or outside reference centers between February 2010 and December 31, 2014. Histological diagnosis was systematically reviewed by expert pathologists from the French Sarcoma Group (FSG). The clinical and histological characteristics and relapse‐free, progression‐free, and overall survival are described with a median follow‐up of 26 months.

### Treatments

Management adhered to local policy without formal recommendation. Surgical procedures included excision (removal of the tumor without normal tissue around tumor), wide resection (removal of the tumor with normal tissue around the tumor), compartmental resection, or amputation. Resection margins were analyzed (R0 microscopically negative margins, R1 macroscopically resected, or R2 incomplete tumor resection). Radiotherapy (RT) and chemotherapy (CT) administration were administered in a neoadjuvant, adjuvant, or palliative setting as chemotherapy. The nature of chemotherapy was classified as “adult protocol” (mainly adriamycin and/or ifosfamide) or “pediatric protocol” (PP) (mainly based on the combination of ifosfamide, vincristine, and actinomycin +/− maintenance chemotherapy with metronomic cyclophosphamide). In the retrospective cohort, all information was available, while in the prospective cohort, the precise description of chemotherapy and radiotherapy was not available. Response status was obtained after completion of treatment (chemotherapy+/− surgery +/− radiotherapy) for each patient. No information was collected on treatment after relapse. Overall survival was recorded in both databases.

### Statistical analysis

Continuous variables were compared using Student's *t*‐test (two‐tailed) and ANOVA if normally distributed or, if not normally distributed, the Mann–Whitney or Kruskal–Wallis tests. Categorical variables were compared using the chi‐square test or Fisher's exact test if necessary.

Median follow‐up was calculated using the inverse Kaplan–Meier method. OS was calculated as the time from the date of diagnosis to the date of the event (death from any cause) or censored at the date of last visit. For patients with localized RMS, relapse‐free survival was calculated as the time from the date of diagnosis to the first relapse (local and/or metastatic) or censored at the date of last visit. OS and relapse‐free survival curves were calculated using the Kaplan–Meier method to assess the probability of survival without event during the follow‐up period and compared using the log‐rank test. Only parameters with a univariate *P*‐value <0.20 were introduced into the multivariate Cox regressions. Proportional hazards assumption was verified on final models by means of Schoenfeld residuals.

Patients with localized RMS (without nodes and metastases) and patients with advanced RMS (with nodes and/or metastases) were separately analyzed. Outcome of patients with locoregional nodal involvement and those with metastasis is similar, so we have gathered both in only one group, called “advanced RMS”. Moreover, patients with localized RMS were analyzed separately according to histological subtypes (alveolar RMS: A‐RMS; embryonal RMS: E‐RMS; and “adult‐type” RMS including pleomorphic, adult spindle cell RMS, and not otherwise specified RMS: P‐RMS).

All tests were two‐sided with *P* < 5% indicating significance. All analyses were performed with SAS 9.3 (Copyright (c) 2002–2010 by SAS Institute Inc., Cary, NC, USA).

## Results

### Patient and tumor characteristics

Table 1 summarizes characteristics of both cohorts. Characteristics differed according to the histological subtype. P‐RMS was more frequently located in the limbs (52/69, 75%) compared with E‐RMS (6/33, 18%) or A‐RMS (18/55, 33%) (*P* = 0.001). The median age was 26 (range 24–76), 25 (range 20–73), and 51 (range 23–86) years for A‐RMS, E‐RMS, and P‐RMS, respectively (*P* = 0.001). The median age was 24 (range 20–76), 22 (range 25–71), 45 (range 18–86), and 38 (range 55–76) years for head and neck (HN) primaries, genitourinary primaries, limbs, or other primary sites, respectively (*P* = 0.001). Lymph nodes (LN) and/or metastases were statistically more frequently noted in A‐RMS (30/55, 54%) compared with E‐RMS (7/33, 21%) or P‐RMS (9/69, 13%) (*P* = 0.001). Only metastases other than LN were observed in 22 of 157 (14%) patients with 13 of 22 (59%) patients with A‐RMS, two of 22 (9%) patients with E‐RMS, and seven of 22 (32%) patients with P‐RMS. Metastasis or LN involvement was significantly associated with the primary site, occurring in nine of 14 (64%) patients with genitourinary RMS, 16 of 39 (41%) with HN RMS, 10 of 76 (13%) with limb RMS, and 11 of 28 (39%) with other sites (*P* = 0.001).

**Table 1:** Patient and tumor characteristics

	Retrospective 1980–2010	Prospective 2010–2015
	*n* (%)	*n* (%)
No of patients	157	292
Gender		
Male	100 (64)	168 (57)
Female	57 (36)	124 (43)
Age (years)		
Median (range)	37 (18–86)	55 (18–99)
18–25	43 (27)	60 (21)
>25	114 (73)	232 (79)
Disease spread		
Localized	111 (71)	204 (70)
Advanced:	46 (29)	74 (30)
N+M−	15	*NR*
N−M+	22	*NR*
N+M+	9	*NR*
Histology		
E‐RMS	33 (21)	49 (17)
A‐RMS	55 (35)	54 (18)
P‐RMS	69 (44)	189 (65)
Site of origin		
Limbs	76 (48)	111 (38)
Head and Neck	39 (25)	65 (22)
Parameningeal	31	*NR*
No parameningeal	8	*NR*
Genitourinary	14 (9)	NR
Vesicoprostatic	6	*NR*
No vesicoprostatic	8	*NR*
Others	28 (18)	116 (40)
Size (cm)		
Median (range)	8 (1–56)	8 (1–25)
<5	31 (20)	74 (25)
≥5	103 (66)	177 (61)
NA	23 (14)	41 (14)

N, nodes; M, metastases; E‐RMS, embryonal rhabdomyosarcoma; A‐RMS, alveolar rhabdomyosarcoma; P‐RMS, “adult‐type” rhabdomyosarcoma.

In the prospective cohort, the proportion of metastatic RMS and histotype distribution was similar to the proportion reported in the retrospective cohort.

### Treatment in the retrospective cohort

In the retrospective cohort, surgery of the primary tumor was performed in 108 of 157 (69%) patients: 89 of 111 (80%) patients with localized RMS versus 19 of 46 (41%) of metastatic patients (*P* < 0.00001). Surgery was described as excision, wide resection, compartmental resection, and amputation in 42/157 (27%), 60/157 (38%), 3/157 (2%), and 2/157 (1%) patients, respectively. Among 96 patients whose surgery was evaluable for margins, R0, R1, or R2 resection was achieved in 58/96 (60%), 28/96 (29%), and 10/96 (10%) of patients, respectively. Surgery was performed for the primary tumor in 67 of 76 (88%) patients with limb RMS, 18 of 28 (64%) with other sites RMS, six of 14 (43%) with genitourinary site, and 17 of 39 (43%) with HN RMS (*P* = 0.0001). Radiotherapy was performed for 107 of 157 (68%) patients including 81 patients with localized RMS. Chemotherapy was administered to 127 of 157 (81%) patients as neoadjuvant (49/157, 31%), adjuvant (37/157, 23%), both (12/157, 8%), or in a palliative situation (29/157, 18%) (Appendix S1). In total, 83 patients who received chemotherapy had localized RMS with the following subtype RMS: 24 of 25 (96%) for A‐RMS, 22 of 26 (84%) for E‐RMS, and 37 of 60 (62%) for P‐RMS (*P* = 0.002). The histological subtype influenced administration of chemotherapy and type of chemotherapy. Patients with A‐RMS, E‐RMS, and P‐RMS received CT for 53 of 55 (96%), 29 of 33 (88%), and 45 of 69 (62%) patients, respectively (*P* = 0.0001). Among 122 patients with a known administered regimen, PP was used for 27 of 51 (53%) with A‐RMS, 20 of 29 (69%) with E‐RMS, and five of 42 (12%) patients with P‐RMS (*P* = 0.0001). Patients older than 25 years received more surgery (*P* < 0.001), less chemotherapy (*P* < 0.001), and less combination of treatments (*P* < 0.003).

### Response to treatment and patterns of failure in the retrospective cohort

Overall, complete remission (CR) after initial treatment was obtained for 93 of 153 (59%) of patients (Table 2).

**Table 2:** Prognostic factor for complete response (CR) in the retrospective study

Variable	No CR *n* = 60 (%)	CR *n* = 93 (%)	*P*
Disease spread (N+ or M+)			
No	29 (48)	80 (86)	<10^−4^
Yes	31 (52)	13 (14)	
Gender			
Female	24 (40)	30 (32)	0.387
Male	36 (60)	63 (68)	
Subtype RMS			
P‐RMS	23 (38)	45 (48)	0.013
A‐RMS	29 (49)	24 (26)	
E‐RMS	8 (13)	24 (26)	
Location			
Limbs	21 (35)	53 (57)	0.008
Head and neck	14 (23)	24 (26)	
Genitourinary	9 (15)	5 (5)	
Other	16 (27)	11 (12)	
Radiotherapy			
No	24 (40)	22 (24)	0.046
Yes	36 (60)	71 (76)	
Chemotherapy			
No chemotherapy	8 (13)	21 (23)	0.210
Nonpediatric protocol	31 (52)	36 (39)	
Pediatric protocol	18 (30)	34 (36)	
NA	3 (5)	2 (2)	
Surgery			
No surgery	34 (57)	13 (14)	<0.001
R > 0	14 (23)	23 (25)	
R = 0	7 (12)	50 (54)	
R = NA	5 (8)	7 (7)	
Tumor size			
<50 mm	5 (8)	26 (28)	0.017
≥50 mm	41 (68)	59 (63)	
NA	14 (23)	8 (9)	
Natural size (mm)	98.7 ± 82.7	81.5 ± 54.8	0.157
Age			
<25	19 (32)	23 (25)	0.360
≥25	41 (68)	70 (75)	
Natural age (years)	41.2 ± 20.7	41.2 ± 17.9	0.999

N, nodes; M, metastases; E‐RMS, embryonal rhabdomyosarcoma; A‐RMS, alveolar rhabdomyosarcoma; P‐RMS, “adult‐type” rhabdomyosarcoma; NA, missing data.

Complete remission was obtained in 80 of 111 (72%) patients with localized RMS and 13 of 46 (28%) patients with advanced RMS (*P* = 0.0001). Predictors for CR are given in Table 2. Evaluation at the end of treatment was CR was obtained at the end of treatment for 50 of 57 (88%) patients who achieved R0 after surgery versus 23 of 37 (62%) patients after R1/R2 resection versus 13 of 47 (27%) patients when surgery was not performed (*P* = 0.0001). With a median follow‐up of 8.5 years, median OS was 49 months (95% CI: 31–284) versus 9 months (95% CI: 9–16) for patients with, respectively, complete response after treatment or not (*P* < 0.0001).

Among the 111 of 157 (70%) patients with localized RMS at diagnosis, data for local relapse were available for 109 of 111 (98%) patients, and 37 of 109 (34%) patients relapsed. Metastases occurred for 45 of 111 (40%) patients. In total, 22 of 109 (20%) patients experienced both local relapse and metastases. Median time to relapse was 9.3 months since initial diagnosis (range: 0.4–199). When LN was present at diagnosis, metastases occurred in nine of 15 (60%) patients at a median time of 8.9 months since initial diagnosis (range: 2.7–13.9).

### Survival in the retrospective cohort

**Figure 1:**
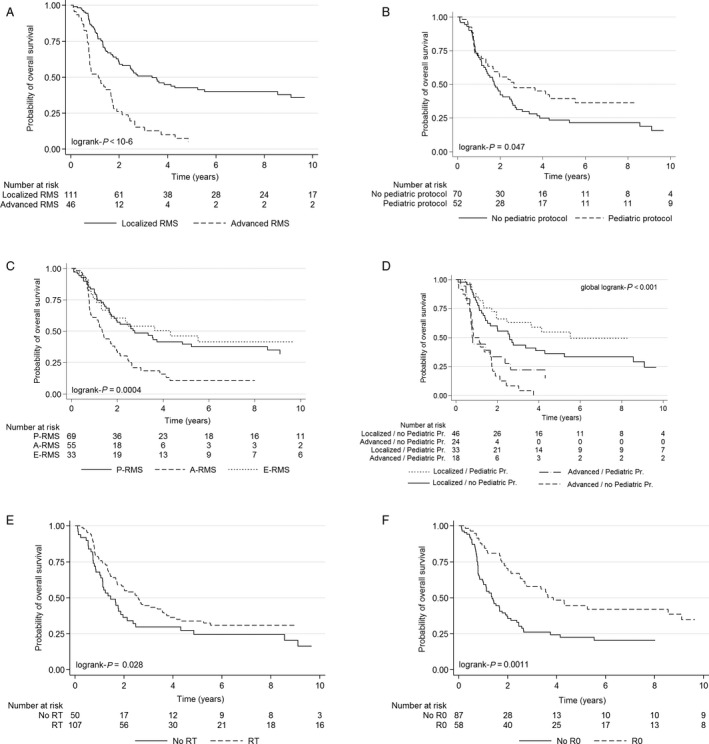
Overall survival for RMS (retrospective study). 
(A) Overall survival for localized and advanced RMS; (B) overall survival and treatment: administration of pediatric protocol; (C) overall survival and histological subtype; (D) overall survival and treatment: administration of pediatric protocol according to stage disease; (E) overall survival and treatment: administration radiotherapy; (F) overall survival and treatment: R0 surgery (R0 versus no R0: R>0 + no surgery).

With a median follow‐up of 8.5 years, 96 of 157 (61%) patients died of disease, including 54 of 111 (49%) with localized RMS and 42 of 46 (91%) with advanced RMS (Tables 3 and 4). Five‐year OS rates for patients with localized RMS and advanced RMS were 43% (median: 40 months, range = 1–337) and 5% (median: 13 months, range 1–297), respectively (*P* < 0.0001) (Fig. 1). Median OS was 16 (range: 2–227), 33 (range = 1–337), and 51 months (range = 1–297) for A‐RMS, P‐RMS, and E‐RMS, respectively. In patients with localized disease, median OS was 24, 42, and 66 months for A‐RMS, P‐RMS, and E‐RMS, respectively. In patients with metastatic disease at diagnosis, median OS was 9, 13, and 28 months for A‐RMS, P‐RMS, and E‐RMS, respectively. In a multivariate analysis, OS of localized RMS was significantly better in patients with non‐A‐RMS (*P* = 0.01), younger age (*P* = 0.004), R0 resection (*P* < 0.0001), receiving radiotherapy (*P* = 0.011), and pediatric chemotherapy protocols (*P* = 0.003) (Fig. 1). For advanced RMS, OS was positively influenced by non‐A‐RMS histological subtypes (*P* = 0.009), R0 resection (*P* = 0.04), and use of RT (*P* = 0.02). In a multivariate analysis, PF for OS in A‐RMS and E‐RMS included gender, location, age, administration of pediatric protocols, and R0 surgery. For P‐RMS, PF included radiotherapy, R0, and age. Predictors for OS are given in Table 3 and Table 4.

**Table 3:** Prognostic factor for overall survival stratified by histological subtypes (retrospective study)

		Univariate analysis for all RMS						Stepwise multivariate analysis for all RMS					
		Localized RMS			Advanced RMS			Localized RMS			Advanced RMS		
		HR	95% CI HR	*P*	HR	95% CI HR	*P*	HR	95% CI HR	*P*	HR	95% CI HR	*P*
Gender	Male vs. Female	1.26	0.73–2.16	0.830	0.79	0.43–1.46	0.453	1.72	0.94–3.15	0.081	–	–	–
Histotype	P‐RMS vs. E‐RMS	1.06	0.56–2.03	0.850	2.65	0.88–7.97	0.083	0.83	0.37–1.88	0.653	9.57	2.24–40.82	0.002
	A‐RMS vs. E‐RMS	1.58	0.77–3.27	0.215	3.43	1.23–9.55	0.018	2.83	1.25–6.40	0.012	5.90	1.55–22.50	0.009
Site	GU/Other vs. Limbs/HN	0.77	0.40–1.47	0.422	1.61	0.88–2.96	0.121				2.41	1.16–4.97	0.018
Radiotherapy	Yes vs. No	0.77	0.45–1.32	0.342	0.67	0.36–1.22	0.190	0.42	0.22–0.82	0.011	0.42	0.18–0.98	0.021
Chemotherapy	No PP vs. no chemotherapy	1.23	0.67–2.25	0.511	1.07	0.25–4.62	0.923	0.76	0.38–1.54	0.455	–	–	–
	PP vs. no chemotherapy	0.71	0.35–1.45	0.347	0.70	0.15–3.16	0.641	0.19	0.07–0.57	0.003	–	–	–
Surgery	>R0 vs. No Surgery	0.59	0.28–1.24	0.162	0.41	0.28–1.24	0.029	0.10	0.04–0.30	<0.0001	0.42	0.18–0.98	0.044
	R0 vs. No Surgery	0.51	0.27–0.98	0.042	0.46	0.27–0.98	0.160	0.06	0.02–0.18	<0.0001	0.25	0.07–0.94	0.041
Size	<50 vs. ≥50	0.44	0.22–0.89	0.021	0.26	0.06–1.09	0.066				–	–	–
Natural age													
Age	<25 vs. ≥25	0.59	0.30–1.16	0.126	1.03	0.56–1.91	0.100	0.23	0.08–0.63	0.004			
Years	>2000 vs. ≤2000	0.95	0.57–1.60	0.850	1.46	0.78–2.75	0.234	–	–	–	–	–	–

		Univariate analysis A/E‐RMS			Univariate analysis P‐RMS			Stepwise multivariate analysis P‐RMS			Stepwise multivariate analysis A/E‐RMS		
		HR	95% CI HR	p	HR	95% CI HR	P	HR	95% CI HR	P	HR	95% CI HR	*P*
Gender	Male vs. Female	1.41	0.64–3.07	0.392	1.16	0.55–2.47	0.692				3.63	1.07–12.31	0.038
Histotype	P‐RMS vs. E‐RMS												
	A‐RMS vs. E‐RMS												
Site	GU/Other vs. Limbs/HN	0.34	0.10–1.12	0.075	1.35	0.61–3.03	0.460				0.11	0.01–0.89	0.038
Radiotherapy	Yes vs. No	1.60	0.61–4.19	0.336	0.46	0.23–0.91	0.026	0.27	0.11–0.71	0.008	–	–	–
Chemotherapy	No PP vs. no chemotherapy	1.38	0.39–4.86	0.617	1.10	0.54–2.25	0.793	1.26	0.50–3.21	0.623	–	–	–
	PP vs. no chemotherapy	0.65	0.18–2.26	0.493	0.31	0.04–2.41	0.265	0.39	0.05–3.34	0.393	0.19	0.06–0.66	0.009
Surgery	>R0 vs. No Surgery	0.77	0.28–2.12	0.607	0.03	0.01–0.28	0.003	0.06	0.01–0.73	0.027	0.18	0.04–0.92	0.040
	R0 vs. No Surgery	0.67	0.29–1.55	0.347	0.02	0.01–0.23	0.001	0.03	0.01–0.36	0.005	0.13	0.03–0.62	0.010
Size	<50 vs. ≥50	0.43	0.18–1.06	0.067	0.37	0.11–1.21	0.099	–	–	–	–	–	–
Natural age					1.04	1.01–1.06	0.004	1.04	1.01–1.07	0.014			
Age	<25 vs. ≥25	0.45	0.21–0.98	0.043	0.73	0.33–1.60	0.429				0.24	0.07–0.84	0.026
Years	>2000 vs. ≤2000	1.13	0.55–2.33	0.731							–	–	–

E‐RMS, embryonal rhabdomyosarcoma; A‐RMS, alveolar rhabdomyosarcoma; P‐RMS, “adult‐type” rhabdomyosarcoma.

**Table 4:** Prognostic factors for relapse in localized RMS from the retrospective series (*n* = 111)

Variable	Univariate analysis			Multivariate analysis		
	HR	95% CI HR	*P*	HR	95% CI HR	*P*
Gender						
Male vs. Female	1.17	0.65–2.09	0.582	2.02	0.97–4.19	0.059
Histotype						
P‐RMS vs. E‐RMS	1.57	0.74–3.33	0.230	0.93	0.34–2.55	0.888
A‐RMS vs. E‐RMS	2.07	0.90–4.75	0.085	3.58	1.15–11.11	0.028
Site						
Genitourinary/Other vs. Limbs/Head and Neck	0.50	0.23–1.11	0.088	0.89	0.35–2.26	0.809
Radiotherapy						
Yes vs. No	0.69	0.38–1.25	0.220	0.31	0.13–0.74	0.008
Chemotherapy						
Nonpediatric protocol vs. no chemotherapy	0.81	0.45–1.50	0.515	0.60	0.26–1.35	0.216
Pediatric protocol vs. no chemotherapy	0.28	0.12–0.64	0.003	0.11	0.03–0.38	0.001
Surgery						
>R0 vs. No Surgery	1.28	0.52–3.18	0.596	0.69	0.22–2.15	0.520
R = 0 vs. No Surgery	1.05	0.45–2.42	0.913	0.26	0.08–0.81	0.021
Size						
<50 vs. ≥50	0.61	0.31–1.19	0.149	0.74	0.30–1.84	0.517
Age at diagnosis						
<25 vs. ≥25	0.53	0.25–1.13	0.101	0.96	0.31–2.97	0.939
Year						
>2000 vs. ≤2000	0.49	0.27–0.90	0.021	0.58	0.28–1.21	0.149

E‐RMS, embryonal rhabdomyosarcoma; A‐RMS, alveolar rhabdomyosarcoma; P‐RMS, “adult‐type” rhabdomyosarcoma.

In a multivariate analysis, PFS of localized RMS was positively correlated with female gender, non‐A‐RMS histologies, use of RT, use of pediatric protocols, and R0 surgery (Table 4).

### Characteristics and outcome in the prospective nationwide cohort

This is the nationwide series of patients with RMS treated in France between 2010 and 2014 and differs therefore from the retrospective series obtained from a selected panel of large centers. As shown in Table 1, an overall higher proportion of patients with P‐RMS was noted in this prospective series.

**Figure 2:**
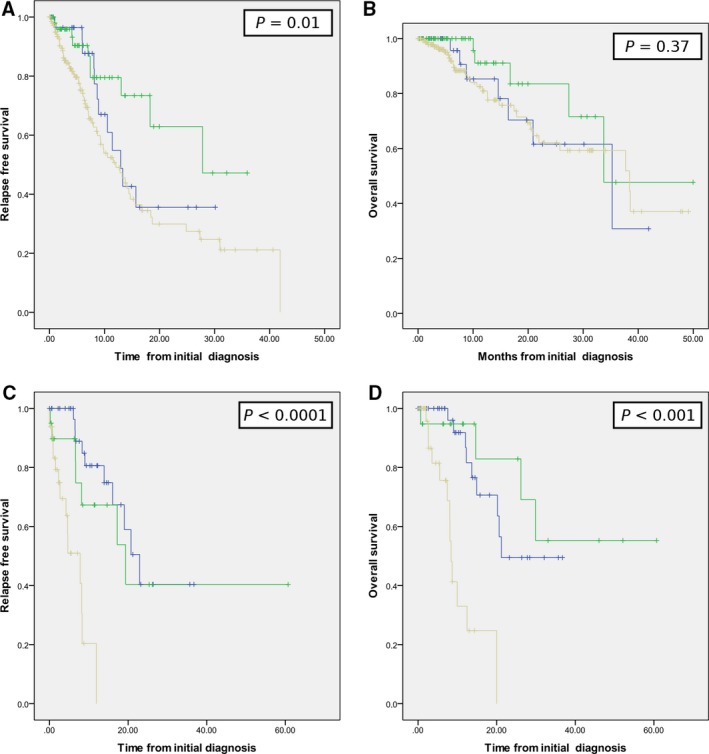
Overall survival and relapse‐free survival for RMS (prospective study). 
(A) RFS of localized RMS. (B) Overall survival of localized RMS; (C) RFS of metastatic RMS. (D) Overall survival of metastatic RMS (green: E‐RMS; blue: A‐RMS; and yellow: P‐RMS).

Median OS was 35 months and 37 months and not reached for A‐RMS, P‐RMS, and E‐RMS, respectively. Median PFS was 19, 11, and 27 months for A‐RMS, P‐RMS, and E‐RMS, respectively. The OS and PFS of E‐RMS were superior in the localized phase (nonsignificant) but was worse compared with patients treated in the 13 reference centers in the previous period for both OS and PFS, with a median PFS < 20 months and median OS < 40 months (Fig. 2). Although the outcome of patients treated in these centers was marginally better (not shown), this finding highlights the severe prognosis of adult RMS in unselected patient populations from both reference and nonreference centers.

## Discussion

Rhabdomyosarcoma is a rare tumor in adult patients. In the prospective cohort, close to exhaustive, there were 292 incident cases of adult RMS in 5 years in this country of 65 million inhabitants for an estimated incidence of 0.9/10^6^/year.

Adult RMS is a difficult‐to‐treat cancer because of its rarity and its heterogeneity. We usually attempt the treated adult A/E‐RMS according to the pediatric guidelines. The present study underlines that pleomorphic RMS is more likely treated as other adult soft tissue sarcomas, with more heterogeneity.

Nevertheless, to improve the standard of care, a better understanding of treatment as performed in routine setting is a logical starting point.

The FSG reports one of the largest recent retrospective studies performed in a multicenter setting at a national level for adult RMS. These results are consistent with those described in the literature. RMS occurs frequently in men and young adults: 37 versus 26 years in other studies and increased by twofold in P‐RMS 11, 12, 13, 14, 15, 16, 17. HN and extremities tumors represent the main primary site in this study and previous reports 11, 12, 16, 17. Synchronous metastases were observed in 20% versus 17–44% in previous studies 11, 13, 14, 15, 16, 17. LN involvement was uncommon (14%) compared with other series (33–46%) 11, 16, 17. This finding is likely related to the prevalence of P‐RMS: 44% versus 9–20% with the exception of the Little series focused on localized RMS 12 (Table 5). In this present work, the clinical presentation is variable according to the histological subtype and is generally worse than that of the pediatric population with RMS.

**Table 5:** Present study and data

Auteur	*N*	Age	Time period	Histological subtype (%)			M+ (%)	OS 5 (%)	OS5 (median) months	OS5 (%)	OS5 (%)
				Embryonal	Alveolar	Pleomorphic NOS		Cohort	Cohort	Localized	M
Lloyd,83 (*MSKCC*)	54	40 (20–73)	1950–1978	100			11	21			17
La Quaglia,94 *(MSKCC)*	290		1970–1991	77	14	9	23	56			
Hawkins,01 (*MSKCC)*	84	23 (16–76)	1982–1999	53	30	17	44	35	22		
Esnaola, 01 *(Dana Farber)*	39	26 (16–82)	1973–1996	18	56	26	33	31			
Little,02 *(MDA)*	82	27 (17–84)	1960–1998	34	23	43	0	44			
Ferrari,03 *(Milan)*	171	27 (19–83)	1975–2001	33	34	32	18	40	38		4
Gerber,13 *(MDA)*	148	27 (19–83)	1990–2011	54	33	14	36	34		45	
Dumont, 13 (MSKCC)	239	10–102 (19)	1957–2003	38	23	37	32			44	18
Present study (retrospective series)	157	37 (18–86)	1980–2010	33	55	69	46	31	24	43	5
Present study (prospective series)	292	55 (18–99)	2010–2015	49	54	189	74		40		

The 5‐year OS is 30% for our population and confirms the poor prognosis of adult RMS (Table 5). For localized RMS, OS is similar to that reported by the MDA and MSKCC experience 14, 15. Importantly, the outcome of patients initially treated in the reference center in the retrospective cohort (Conticabase series) is superior to that of the nationwide and more recent series, possibly explaining the excellence of the reference center compared with other centers 18. The relapse rate is consistent with that reported in the literature: 33–57% local relapses 11, 12, 13, 14, 19 and up to 48–68% metastatic relapse 14, 16, 19. It is observed often during the first year, leading to death in 6–12 months 11, 12, 14, 15, 16, 17, 19, with median survival of 9, 8, and 7 months for patients with local relapse, metastatic relapse, or both in the present work. This outcome is notably inferior to that achieved in other ASTS of adults in 2016. The present report is one of the largest studies and confirms the poor prognosis of adult RMS treated in reference centers or in the general population of patients with RMS.

The univariate analysis and multivariate analysis of clinical and therapeutic PF performed only in the Conticabase series enable us to conclude that adults with RMS share the same PF in terms of disease characteristics compared with pediatric patients. For therapeutic parameters, surgery, radiotherapy, and chemotherapy are identified as PF for OS. But in our study, patients older than 25 years received significantly less chemotherapy and numerous treatments. Surgical results were consistent with those previously reported 11, 12, 13, 15, 16, 17, 18, 19. The absence of surgery and incomplete resection were deleterious for OS. Adjuvant RT and PP were also associated with a better outcome. This finding is consistent with the nationwide French NetSarc reference work that observed a twofold increased R2 surgery outside the NetSarc center and threefold more secondary resection after primary surgery performed without multidisciplinary tumor board in other sarcoma types 18. In the present study, local surgery had a favorable impact on survival even for patients with metastatic RMS which is consistent with the MSKCC and French pediatric experience. These observations could change this approach, and advanced disease surgery should be debated whenever possible 15, 20. Radiotherapy also had a favorable impact on OS for localized and metastatic disease addressing the issue of local treatment in a metastatic setting.

A univariate analysis and multivariate analysis were also performed for each histological subtype in localized RMS. For A‐RMS and E‐RMS, age, location, surgery R0, and PP are relevant. These factors are similar to those reported in the pediatric patients. The positive impact of PP for A‐RMS and E‐RMS on OS is also reported by MSKCC and MDA experience 14, 15 and Ferrari who described better survival with administration of chemotherapy per current guidelines for pediatric RMS 11. This finding suggests that PP should be considered for adult patients with A‐RMS and E‐RMS. Conversely, for nonmetastatic P‐RMS, chemotherapy was not associated with an improved OS in our study. The data presented do not support the use of pediatric regimen in P‐RMS but clearly cannot allow to draw a definitive conclusion on this question, which could be obtained only from a prospective study. It is still important to highlight that P‐RMS are not as responsive (in terms of CR/PR rates) as A/E‐RMS to CT. The best combination of CT for P‐RMS is not defined. There are now a large number of adult series all showing results achieved with doxorubicin‐containing regimens, and because this is the largest evidence in terms of patient numbers, it is reasonable to infer that doxorubicin remains the standard of care. The situation is different for A/E adult RMS, while their management is similar to the pediatric populations for most patients, the level of evidence for adult patient population is not so clear (as for pediatric population), in the absence again of any randomized trial. Considering the toxicity observed in this retrospective series, firm recommendations cannot be proposed; if considered feasible by the adult physician, pediatric regimens and strategies are certainly a relevant option, in particular for young adults, but may be not applicable to older patients, where adult type of regimens (AI based or A can be safely applied). Prospective and homogeneous management need to be implemented and analyzed to identify how adult can be treated with A/E‐RMS if pediatric protocol is not feasible (specifically after 30 years old).

However, the recent ISG/Eurosarc suggested an improved survival achieved with neoadjuvant CT in high‐risk patients 21, 22, 23, 24, 25, 26, 27. Chemotherapy is still a matter of debate and must be discussed in a dedicated multidisciplinary committee with an individual approach basis 28. For all adult sarcoma in a localized phase, R0 surgery remains the cornerstone of treatment 29, 30, 31, 32, 33, 34, 35. The present study underlines the heterogeneity of adult RMS management. This heterogeneity is a study limitation, but at the end is helpful to identify factors favorably influence the outcome. Another study limitation is that we are not able to analyze the prognostic value of the presence (or absence) of specific translocation; nevertheless, the vast majority of adult RMS are undifferentiated ones without specific translocation. We aim to use the prospective cohort to validate the prognostic factors identified in the retrospective series; nevertheless, this requires more mature data and longer follow‐up.

Why are adult patients with A‐RMS and E‐RMS at higher risk of relapse than children affected with the same disease? Since 1972, within IRS group trials, 5‐year OS of children has improved from 55%, 63%, and 69% to 73% in an IRS IV study, representing a threefold increase over these 30 years 6, 7, 8, 9, 36. This improvement did not translate to adult patients, and several hypotheses are suggested involving age, clinical presentation, and intensity/duration of treatment. Ferrari et al., and more recently, the MDA study, reported that only the oldest patients treated with a non‐PP had less favorable survival 11, 14. Clinical presentation is also less favorable. All retrospective studies note more aggressive disease with more unfavorable sites 1, 36, 37, 38, 39, more LN, more metastasis 9, 36, 40, 41, and more A‐RMS. Nevertheless, all parameters being equal, the outcome of adult patients with RMS is less favorable. Beyond the obvious difference in therapeutic approaches and management, the genomics of A‐RMS and E‐RMS should be compared between adult and children, similar to that performed for synovial sarcoma 42. Sharing the same classification would allow easier comparison between clinical presentation and behavior in adults and children. Finally, specific chemotherapy for A‐RMS and E‐RMS is an independent prognostic value in our study and others 2, 6, 7, 8, 9, 11, 14, 15, 38, 39, 40, 41, 43, 44, 45, 46, 47. North American and European pediatric groups have identified several risk factors (favorable location, IRSG stage, IRSG group, and age) to define the clinical group and deliver a regimen adapted to the risk group. Thus, reflection should be engaged for common protocols, and PP should be adjusted for risk groups using vincristine, dactinomycin, and alkylating agents 6, 7, 8, 9, 10, 48, 49, 50, 51, 52, 53, as anthracycline is always controversial in PP. For patients up to 50 years old, toxicity in PP is unacceptable with age, as demonstrated through OS or Ewing protocols.

## Conclusion

This is the largest study analyzing all factors in univariate analysis and multivariate analysis for localized and metastatic disease and for each histological subtype. Specific management for A‐RMS and E‐RMS using a pediatric protocol chemotherapy and carcinologic surgery is the cornerstone to improving survival. The FSG experience emphasizes the urgent need to build a worldwide clinical trial using these rare entities that exhibit a dismal prognosis.

## Conflict of Interest

None declared.

## Supporting information


**Appendix S1.** Regimens of chemotherapy.
