# Supplementary material for: Outcome of 449 adult patients with rhabdomyosarcoma: an observational ambispective nationwide study
Source: Cancer Med. 2018 Jun 28;7(8):4023–35. doi: 10.1002/cam4.1374 (PMC6089183; doi:10.1002/cam4.1374)
Supplement: Supplementary file 1 — Appendix S1. Regimens of chemotherapy. [file CAM4-7-4023-s001.docx]

**Appendix S1.** Regimens of chemotherapy

| **Regimens** | **N total = 127** | **Agents** |
| --- | --- | --- |
| **No Pediatric protocol** | **70 (55)** |  |
| A | 9 (13) | Adriamycin |
| MAI | 23 (33) | Adriamycin ifosfamide |
| MAID | 20 (28) | Adriamycin ifosfamide dacarbazine |
| MAIDO | 1 (1.4) | Adriamycin ifosfamide dacarbazine oncovin |
| API | 4 (6) | Adriamycin cisplatin ifosfamide |
| TNF-melphalan | 1 (1.4) | TNF-melphalan |
| CYVADIC | 4 (6) | Cyclophosphamide vincristine adriamycin dacarbazine |
| AD | 3 (4) | Adriamycin dacarbazine |
| EP | 1 (1.4) | Etoposide platine |
| IV | 1 (1.4) | Ifosfamide vincristine |
| Trophosphamid | 1 (1.4) | Trophosphamid |
| DECAV | 1 (1.4) | Ifosfamide adriamycin dacarbazine vindesin platin |
|  | 1 (1.4) | Adriamycin vindesine ifosfamide cisplatin |
| **Pediatric protocol** | **52 (41)** |  |
| IVA | 9 (1.1) | Ifosfamide vincristine adriamycin |
| IVAc | 14 (27) | Ifosfamide vincristine actinomycin |
| IVA/IVADo | 6 (11.5) | Ifosfamide vincristine actinomycin doxorubicin |
| MAIDO/IVA/CEV | 2 (3.8) | Ifosfamide vincristine actinomycin adriamycin |
| IVAD | 8 (15.3) | Ifosfamide vincristine adriamycin dacarbazine |
| VIDE | 2 (3.8) | Vincristine ifosfamide doxorubicin etoposide |
| IVA/IVE/CEV | 3 (5.7) | Ifosfamide vincristine actinomycin etoposide carboplatin epirubicin |
| Protocole SIOP/MMT | 6 (11.5) |  |
| IVA/CEV | 2 (3.8) | Ifosfamide vincristine actinomycin carboplatin epirubicin |
| **NA** | **5 (3)** |  |
